# Tract Profiles of White Matter Properties: Automating Fiber-Tract Quantification

**DOI:** 10.1371/journal.pone.0049790

**Published:** 2012-11-14

**Authors:** Jason D. Yeatman, Robert F. Dougherty, Nathaniel J. Myall, Brian A. Wandell, Heidi M. Feldman

**Affiliations:** 1 Department of Psychology, Stanford University, Stanford, California, United States of America; 2 Stanford Center for Cognitive and Neurobiological Imaging, Stanford University, Stanford, California, United States of America; 3 Stanford University School of Medicine, Stanford, California, United States of America; 4 Division of Neonatal and Developmental Medicine, Department of Pediatrics, Stanford University School of Medicine, Stanford, California, United States of America; University of Alberta, Canada

## Abstract

Tractography based on diffusion weighted imaging (DWI) data is a method for identifying the major white matter fascicles (tracts) in the living human brain. The health of these tracts is an important factor underlying many cognitive and neurological disorders. *In vivo*, tissue properties may vary systematically along each tract for several reasons: different populations of axons enter and exit the tract, and disease can strike at local positions within the tract. Hence quantifying and understanding diffusion measures along each fiber tract (Tract Profile) may reveal new insights into white matter development, function, and disease that are not obvious from mean measures of that tract. We demonstrate several novel findings related to Tract Profiles in the brains of typically developing children and children at risk for white matter injury secondary to preterm birth. First, fractional anisotropy (FA) values vary substantially within a tract but the Tract FA Profile is consistent across subjects. Thus, Tract Profiles contain far more information than mean diffusion measures. Second, developmental changes in FA occur at specific positions within the Tract Profile, rather than along the entire tract. Third, Tract Profiles can be used to compare white matter properties of individual patients to standardized Tract Profiles of a healthy population to elucidate unique features of that patient's clinical condition. Fourth, Tract Profiles can be used to evaluate the association between white matter properties and behavioral outcomes. Specifically, in the preterm group reading ability is positively correlated with FA measured at specific locations on the left arcuate and left superior longitudinal fasciculus and the magnitude of the correlation varies significantly along the Tract Profiles. We introduce open source software for automated fiber-tract quantification (AFQ) that measures Tract Profiles of MRI parameters for 18 white matter tracts. With further validation, AFQ Tract Profiles have potential for informing clinical management and decision-making.

## Introduction

A major goal of clinical neuroimaging research is to make measurements that can accurately diagnose or characterize clinical conditions and predict clinical outcomes. Achieving the goal requires an efficient procedure to (1) identify equivalent brain structures in healthy controls and individual patients and (2) measure biological properties of the structures that are sensitive to clinical abnormalities. In this report, we introduce an automated method for identifying specific white matter fascicles from diffusion weighted imaging data and quantifying biological properties along the length of these fascicles.

Diffusion weighted imaging (DWI) is a magnetic resonance imaging (MRI) method that measures water diffusion in brain tissue in multiple directions. Water diffusion probes tissue organization at the micrometer scale within an MRI voxel. In regions of cerebral spinal fluid (CSF), the mean displacement of water molecules due to diffusion is similar and relatively large in all directions (isotropic). In gray matter, cell membranes hinder the movement of water molecules, and therefore the mean displacement of water molecules is smaller but still isotropic. In white matter, myelinated axons are directionally coherent causing anisotropic diffusion that is much smaller perpendicular to the axons than it is parallel to the axons [1]– for review see [4].

The mean rate of water diffusion is measured by the apparent diffusion coefficient (ADC). In white matter the ADC is greatest parallel to the principal orientation of an axon bundle (fascicle/fiber tract) and is reduced and often approximately constant in all the directions perpendicular to the principal direction. Summary measures such as fractional anisotropy (FA) can be derived to infer how restricted diffusion is perpendicular to the primary orientation of the fascicle. Diffusion properties are routinely used for group comparisons between clinical populations and control groups to infer the neurobiology of the disease.

One approach to the analysis of diffusion-weighted imaging is voxel-based analysis. This method computes statistics independently for the diffusion properties of each voxel within the brain image. To assure that the same voxels are compared across subjects, coregistration algorithms are used to align the brain images of the subjects to a common coordinate frame. Particularly for patient populations, voxel-based analysis does not have sufficient precision at the individual level because the shape of long-range fiber tracts varies substantially among subjects [5], [6]. Coregistration algorithms used in voxel-based analysis do not accurately align fiber tracts due to variation in tract size and shape [5]. Methods such as Tract-Based Spatial Statistics (TBSS) that compute statistics on “voxel skeletons” may be an improvement over conventional voxel-based analysis. However, like any voxel based technique, TBSS cannot ensure that any voxel corresponds to the same tract across participants and there is only modest agreement between TBSS-based tract definitions and the actual location of a tract in an individual's brain [7].

Tractography algorithms use estimates of the principal diffusion direction (or orientation distribution function) to trace the continuous trajectory of white matter fascicles through a three-dimensional brain volume [8], [9]. Tractography is widely considered the most accurate method for identifying the white matter fascicles in the living human brain, and has been validated in artificially constructed fiber structures (called phantoms), animals and humans [10]–. These algorithms have even proven useful at the individual level for identifying the location of essential fiber bundles during neurosurgery [13]–[16].

One limitation of tractography for large-scale clinical research and time-sensitive clinical practice is that the usual methods for identifying major fascicles are laborious and time consuming. They typically rely on manually drawing regions of interest (ROIs) that disambiguate the trajectory of a known fascicle from other fascicles within the brains of each participant [17], [18]. There is much interest in developing methods that rapidly and reliably identify and measure fiber tracts in an individual's brain, and there has been recent progress in automating tract identification [19].

A second limitation of tractography is that diffusion properties are typically averaged over the entire length of the white matter tract. However, diffusion measurements vary along the tract trajectory [6]. One reason for this variation is the presence of crossing tracts that lower FA at the tract juncture. Equally important, axons do not always run the entire length of a fascicle, and in many cases different neural populations enter and exit at different points along the fascicle. Hence, averaging along the entire tract may obscure potentially important information. Mean diffusion properties for a tract generally change during development [20] and there are group differences in a variety of clinical conditions [4], [21], [22]. When there is a change in the mean for a tract, it is possible that the change is reflected throughout the entire tract or that the change is driven principally by a small region within the tract [6], [23]. Mean measures are not sufficiently sensitive to classify an individual's level of development or clinical outcome. If the key axons arise from a population that passes through only a portion of the fascicle, then measures that focus just on that portion will be far more sensitive than averaging across the length of the fascicle. Ideally an analytic method for clinical research and practice would capitalize on the precision of tractography for localizing fiber tracts in individual brains and simultaneously preserve information about the diffusion measurements at different locations on the tracts. Recent reports have emphasized the utility of analyzing diffusion properties along the tract trajectory in healthy brain anatomy [24], [25], development [26], aging [27], and clinical conditions [28]–[32].

In this paper, we present a framework for quantifying diffusion measurements at multiple locations along the trajectory of a white matter tract, creating a “Tract Profile” of diffusion measurements. To create Tract Profiles reliably and efficiently, we introduce an algorithm that automatically identifies 18 major white matter tracts in healthy and diseased brains and makes measurements at anatomically equivalent locations along their trajectories. We call the software Automated Fiber Quantification (AFQ), which we make open source and freely available. The applications in this paper elucidate the value of Tract Profiles for scientific investigation, clinical research and practice.

The first aim of this study is to demonstrate the systematic variation in diffusion properties along the trajectory of 18 fiber tracts within both hemispheres. For a group of typically developing children ages 9 through 16, we demonstrate that the Tract FA Profiles are reliable and consistent.

The second aim is to compare the Tract Diffusion Profiles as a function of age. We show that Tract FA Profiles change with age, and that the changes in FA occur at specific locations within each tract.

The third aim is to compare Tract Diffusion Profiles for individual patients with normative or standardized Tract Diffusion Profiles, derived from a healthy age-matched sample. We chose to focus on children born preterm due to the heterogeneity of white matter properties and neuro-developmental abnormalities of that population. Recent research has documented that children born preterm have diffuse white matter injuries. The cause appears to be the vulnerability of oligodendrocyte precursors, the cell line that ultimately produces myelin, between 24 and 32 weeks gestation [33], [34]. We show that Tract Diffusion Profiles identify distinct abnormalities in individual patients that can be linked to the patient's clinical characteristics.

The fourth aim is to use Tract Diffusion Profiles to predict behavioral outcomes in the preterm sample. Reading impairments are common in children born preterm and are thought to result from perinatal white matter injury [33], [35]–[37]. We demonstrate that in the preterm sample reading proficiency is correlated with FA values at specific locations within two tracts: the left arcuate fasciculus and left superior longitudinal fasciculus.

## Results

### Diffusion properties vary systematically along major fascicles

The diffusion properties of a tract can be represented with a vector of measurements sampled at equidistant locations along the tract. We label the vector of diffusion measurements for a tract as the “Tract Diffusion Profile”.

**Figure 1:**
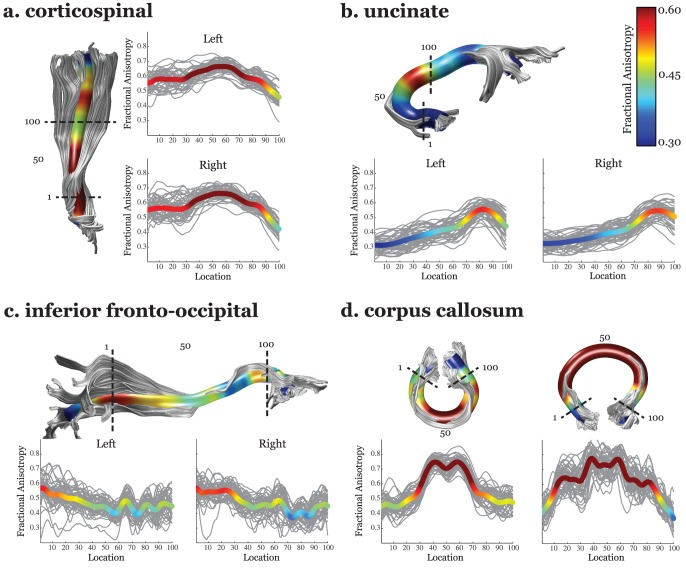
Tract Fractional Anisotropy (FA) Profiles of four major fascicles. (a) cortico-spinal tract, (b) uncinate fasciculus, (c) inferior fronto-occipital fasciculus, and (d) corpus callosum. For each tract, a three-dimensional rendering derived from the Automated Fiber tract Quantification (AFQ) software is shown for a single representative 12-year old female. It indicates the defining Regions of Interest (ROIs) as dotted lines and includes the core or mean fiber, represented as a 5 mm radius tube color-coded based on the FA value at each point along the tract for that subject. Adjacent to the rendering, Tract FA Profiles for left and right hemispheres show the FA along the core fiber (y-axis) at each of 100 equidistant points (x-axis) along the fascicle between the defining ROIs for typically developing children and adolescents age 9 to 16 years old (N = 48). The group mean is shown as a bold line, colored-coded based on the group mean FA value at that point. Tract FA Profiles show a consistent pattern of peaks and valleys of FA across individuals.

We used Automated Fiber Quantification (AFQ) software to generate Tract Diffusion Profiles for a sample of healthy and typically developing participants ages 9 through 16 years (n = 48) (see Methods). In this report we focus on FA but other measures can be examined as well. We found that FA varies systematically along the trajectory of each white matter fascicle. Figure 1 shows the Tract FA Profiles for 48 typically developing children on four tracts in the left and four tracts in the right hemisphere. Examination of Figure 1 demonstrates that subjects reliably show decreases and increases in FA at equivalent locations along the tracts. This variation in FA can be explained by anatomical features of the tracts: geometric properties of the tract, such as curvature; partial volume effects with neighboring structures; and the admixing of crossing, branching, merging or kissing fibers from other fiber tracts.

Below we discuss the anatomical characteristics of the four tracts to explain the peaks and valleys in the Tract FA Profiles.

#### Corticospinal Tract (CST)

The CST shows a dramatic reduction in FA at an equivalent location in all individuals and at that point FA falls to a similar level in each subject. The CST ascends from the brainstem, paralleling the ventricles to the cortex. FA for the CST starts off relatively low due to partial voluming in the brain stem. FA peaks roughly half way between the two defining ROIs, at the level of the internal capsule. At this location fibers are coherently oriented inferior-superior. FA then declines substantially at the level of the centrum-semiovale, a location where callosal fibers cross medial to lateral through the CST and superior longitudinal fasciculus (SLF) fibers cross posterior to anterior through the CST [38].

#### Uncinate Fasciculus

The uncinate fasciculus shows a single peak in FA that consistently occurs in the same location in every subject. From the anterior temporal lobe the uncinate travels in a posterior-medial direction, curves behind the insula, and continues in a superior and anterior direction toward the orbitofrontal cortex. FA for the uncinate fasciculus remains low in the temporal lobe, through the curved portion of tract, and starts steadily increasing when it joins up with the external/extreme capsule and heads anterior towards its frontal lobe end points.

### Inferior Fronto-Occipital Fasciculus (IFOF)

The IFOF shows three distinct and consistent peaks and valleys in its FA profile. The IFOF courses from the occipital lobe through the external/extreme capsule to the inferior frontal cortex. FA is high in the occipital and temporal lobes and declines as the tract heads anterior. The first valley in the Tract FA Profile occurs where the tract bundles together and enters the external/extreme capsule. FA remains low through the initial section of the external/extreme capsule where the tract is thin and intermixed with neighboring gray matter. FA increases where the IFOF merges with the uncinate at the location of the FA peak on the uncinate. The next FA valleys occur in regions where the tract again abuts gray matter or curves and increases where the tract enters regions of thicker white matter where there is less partial voluming.

#### Corpus Callosum, Forceps Major and Forceps Minor

The corpus callosum shows a dramatic two fold decrease in FA as the fibers traverse away from the mid-sagittal plane. The forceps major connects homologous regions of the occipital lobe in each hemisphere and the forceps minor connects homologous regions of the anterior frontal lobe in each hemisphere. FA for both callosal segments is high (∼0.8) near the mid-sagittal plane where callosal fibers are tightly bundled together and coherently organized in the medial-lateral direction. FA decreases substantially as fibers start diverging toward their specific cortical destinations. The forceps major shows a sharp FA decline in homologous regions of the left and right hemisphere. This FA valley occurs where callosal projections merge with longitudinally oriented projections to the occipital lobe. FA then increases slightly as the callosal fibers align with these longitudinal projections destined for occipital cortex.

#### Standardized tract diffusion profiles

**Figure 2:**
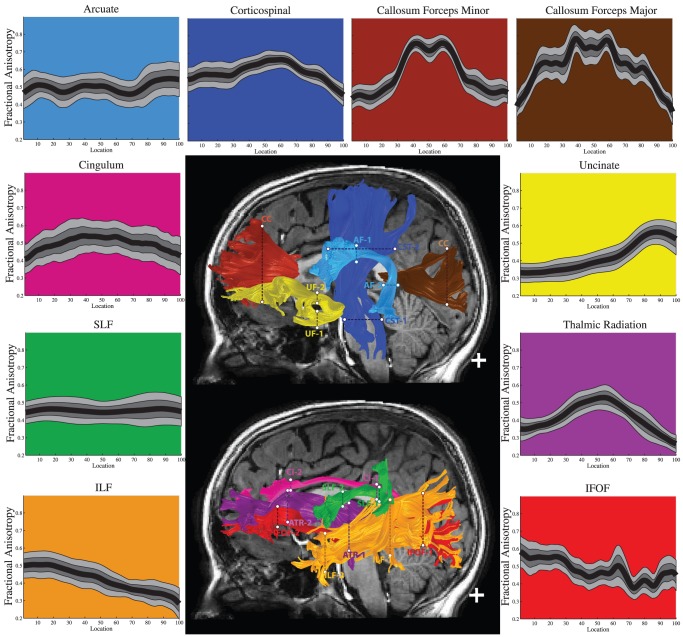
Standardized Tract FA Profiles for 10 tracts in typically developing children and adolescents. In the center, two sagittal T1 images show renderings of 10 major tracts, each with a different color, including the two defining regions of interest (ROIs), marked by dotted lines. Around those images are the Standardized Tract FA Profiles, color-coded to match the tracts in the central image, with FA values plotted for 100 equidistant locations between the two defining ROIs. The black line in each plot represents the mean FA for each point. The dark gray band shows 25^th^ and 75^th^ percentiles and the light gray band shows the boundaries of the 10^th^ and 90^th^ percentiles. (SLF = Superior Longitudinal Fasciculus, ILF = Inferior Longitudinal Fasciculus, IFOF = Inferior Fronto-Occipital Fasciculus).

Given the consistency of Tract FA Profiles across the healthy and typically developing children, it is possible to create a standardized Tract FA Profile for each tract that characterizes the mean and variation of the measure at each point along the tract. Figure 2 shows the mean Tract FA Profiles and the 10^th^ and 90^th^ percentiles for 8 left hemisphere tracts and 2 callosal segments identified by AFQ for the sample of healthy typically developing children. The profiles of right hemisphere pathways were similar. Creating normative Tract Profiles allows for point-wise quantification of tract abnormalities in clinical populations or at-risk individuals that have been scanned with the same MRI sequence.

### Developmental changes in Tract FA Profiles

During development FA increases. The reasons are likely to do with the increase in myelination and directional coherence of the axons. It is known that the rate of maturation varies between tracts [20]; however, it is not known if all locations within a tract change at comparable rates.

**Figure 3:**
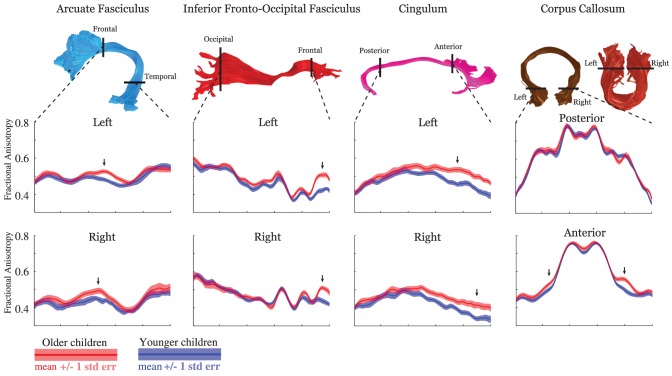
Development of Tract FA Profiles between childhood and adolescence. Standardized Tract FA Profiles for three left and right hemisphere tracts and the posterior and anterior segments of the corpus callosum in younger participants (n = 24, mean age 9.8 years sd = 1.4), represented in blue, and older typically developing children (n = 24, mean age 14.3 years sd = 1.1), represented in red. Renderings of each tract indicate the defining regions of interest. Each plot shows the mean Tract FA Profile +/−1 standard error of the mean confidence interval for each group. Differences in FA across groups occur at specific locations on the Tract FA Profiles. Arrows indicate on the area of the Tract FA Profile showing the greatest group difference (discussed in main text).

To determine whether FA develops throughout the entire tract or only at selected locations, we used a median split to divide the sample of healthy controls into a group of 24 younger subjects (mean age = 9.8, sd = 1.4) and a group of 24 older subjects (mean age = 14.3, sd = 1.1). We used AFQ to compare Tract FA Profiles in the younger and older groups. Figure 3 shows the mean Tract FA Profiles for the younger and older children with a +/−1 standard error of the mean confidence interval computed at each location along the tract.

We found that the Tract FA Profiles had a similar shape for the younger and older children, confirming that the anatomical features that cause variation in FA are in place by age 9 years. However, Tract FA Profiles revealed that changes in FA were not uniform along the tracts. The older children had higher FA than the younger children at specific locations on the Tract Profiles of 15 out of the 18 tracts. The locations of FA development and the locations of FA stability were consistent across the right and left hemisphere. These results add substantial specificity to previous reports of increasing FA as a function of age [20]. While voxel-based analyses typically find varying levels of statistical significance across the brain, voxel-based analysis cannot be used to compare rates of development at different locations on a tract because of substantial spatial variation in statistical power over the brain volume [39] and TBSS voxel skeleton [40].

Below we discuss the anatomical locations of FA development of four tracts in each hemisphere.

#### Arcuate Fasciculus

Mean FA for the entire left and right arcuate was not significantly different between older and younger children. However, FA was significantly higher in the older children at a specific location medial to the central sulcus (marked by an arrow in figure 3a) in the left hemisphere (FA older children = 0.51, FA younger children = 0.46, t = 3.64, p<0.05 corrected) and approached significance at the same location in the right hemisphere (FA older children = 0.48, FA younger children = 0.44, t = 2.13, p = 0.04 uncorrected). At this location, anterior to the principal arc of the tract, fibers are straight and coherently bundled together. We think that this location represents the purest measure of white matter microstructure for the arcuate, uncontaminated by curving and crossing fibers. The increased sensitivity of the Tract FA Profile to developmental change compared to a single tract summary measure illustrates the sensitivity of this methodology to group differences.

#### IFOF

The mean FA for the entire IFOF was greater for older versus younger children; the magnitude of change was small (Left ILF: younger = 0.46, older = 0.48, t = 2.65, p<0.05; Right ILF: younger = 0.46, older = 0.47, t = 0.72, p = n.s.). However, there was a substantial FA increase for the older subjects localized to the frontal lobe portion of the tract (Left IFOF FA: older = 0.50, younger = 0.42, t = 5.72, p<0.05 corrected, Right IFOF FA: older = 0.50, younger = 0.42, t = 4.56, p<0.05 corrected. Location is marked by an arrow in Figure 3b). These findings are consistent with other data that suggests that frontal lobe white matter develops later than occipital and temporal lobe white matter [41], [42].

#### Cingulum

For both the left and the right cingulum, the posterior third of the tract had equivalent FA levels for older and younger children. Differences emerge, and grow in magnitude toward the anterior portions of the tract in both hemispheres (Left cingulum FA: older = 0.49, younger = 0.41, t = 4.15, p<0.05 corrected; Right cingulum FA: older = 0.41, younger = 0.34, t = 3.11, p<0.05 corrected. Location is marked by an arrow in Figure 3c). These findings are also consistent with other data suggesting that white matter of the frontal lobe develops later than posterior regions.

#### Corpus Callosum

There was not significant FA development in the mid sagittal plane. There was also no developmental change in the forceps major. For the forceps minor FA was significantly higher in the anterior frontal lobe portion of the tract in the left hemisphere (older = 0.55, younger = 0.50, t = 3.48, p<0.05 corrected) and approached significance in the right hemisphere (older = 0.54, younger = 0.50, t = 2.43, p = 0.019 uncorrected).

### Detection of neurodevelopmental abnormalities from preterm birth

Children born preterm are at substantial risk of white matter injury. However in this population the mechanisms of injury are variable. Studies of preterm infants report heterogeneous outcomes including diffuse abnormalities in the myelination process due to damage to oligodendrocyte precursor cells, focal necrotic lesions due to severe hypoxic/ischemic insult, and typical white matter development despite neonatal complications [35].

In a previous analysis using an alternate method for analyzing white matter properties, as a group the children born preterm in this sample did not show reductions in FA compared to the controls [43]. These findings are consistent with other reports in the literature and indicate the heterogeneity of brain development after preterm birth [44].

**Figure 4:**
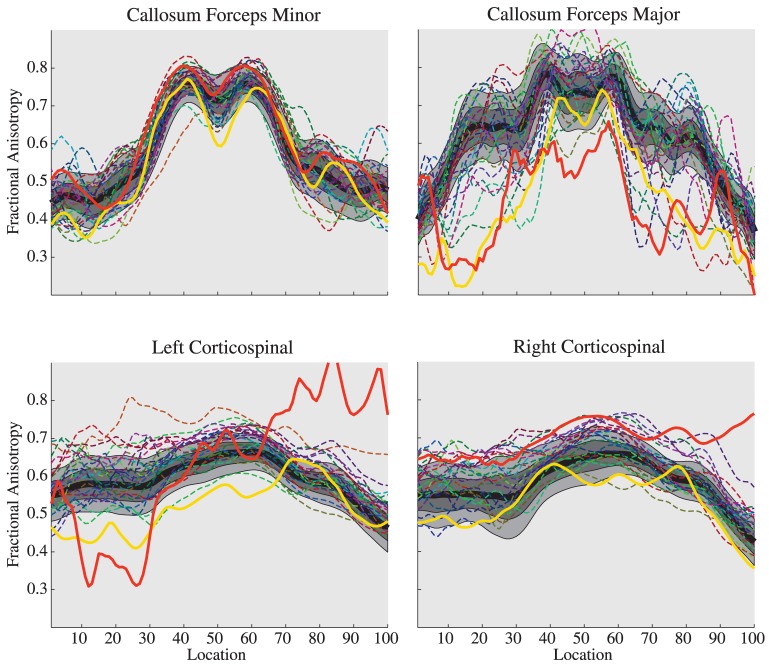
Individual Tract FA Profiles for children born preterm compare to Standardized Tract FA Profiles for typically developing children. For four tracts, Tract FA profiles of individual preterm patients are plotted as a dashed line. Each patient is a different color. Notice the substantial variation in Tract FA profiles, particularly in the callosum forceps major and left corticospinal tract. Two patients (red and yellow solid lines) have unusual Tract FA profiles that correspond to clinical findings (discussed in main text).

We applied AFQ to generate Tract FA Profiles for individual children in the preterm group and to quantify tissue properties along white matter fiber tracts within these individuals relative to reference norms. Figure 4 shows Tract FA Profiles for the forceps major, forceps minor and CST for children born preterm (n = 26) relative to the control group norms. We found wide variation of FA within the group of children born preterm in relation to the norms. Some of the children born preterm have substantial reductions in FA, some have normal FA and some have substantial increases in FA relative to the norms.

These data demonstrate that there is not a canonical neurodevelopmental abnormality that characterizes the whole sample of children born preterm. However when we use a threshold for values outside the typical range (either 10^th^ and 90^th^ or 5^th^ and 95^th^ percentiles) to identify outliers with abnormal Tract Profiles, significantly more patients are identified as outliers than are controls (Χ^2^ (1,*N* = 75) = 5.13, p<0.05). 40% of the patients compare to 16% of controls are identified as outliers on one or more tracts when the 5^th^ and 95^th^ percentile bands are used to define abnormal Tract Profiles.

Evaluating individual Tract FA Profiles we identified two patients with severe abnormalities. Patient #1, shown as a solid red line in Figure 4, has extremely low FA for the forceps major but has extremely high FA along the superior portion of the left and right CST. This patient has severe ventricular dilation. Patient #2, shown as a solid yellow line, has reduced FA for the left CST but normal FA for the right CST. This patient has cerebral palsy that is more severe for the right side of the body. We discuss these patients in detail below.

#### Patient 1: Ventricular Dilatation

**Figure 5:**
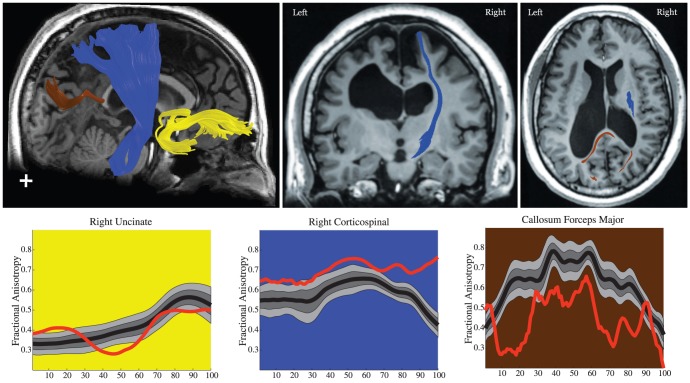
T1 images, tractography results and Tract FA Profiles for Patient 1, a child with ventricular dilatation. In the plots, the black line represents the mean FA for typically developing children at 100 points along the tract and light gray region represents the boundaries for the 10^th^ to 90^th^ percentile. The red line represents the patient's FA along the tract. The patient has variable FA in the uncinate, increased FA along the corticospinal tract and decreased FA in the corpus callosum forceps major.

Patient 1 was a 14-year-old male born prematurely at 27 weeks gestational age with non-shunted ventricular dilatation secondary to grade III–IV intraventricular hemorrhage. Figure 5 shows a T1 weighted anatomical image of patient #1 and three fiber tracts identified in the patient with AFQ; the right uncinate fasciculus, the right cortico-spinal tract and the forceps major of the corpus callosum. The child showed normal diffusion properties along the uncinate fasciculus, a tract that is spatially separated from the ventricles. The child has thinning of the corpus callosum and low FA along the full trajectory of the forceps major. This finding is consistent with other studies of children born prematurely. To test whether the reduced FA could be accounted for by partial voluming with CSF in this patient with enlarged ventricles we examined the patient's Tract Mean Diffusivity (MD) Profile. MD values were elevated at node 20, but otherwise MD values were within the normal range indicating that there was not a substantial change in the water content of forceps major voxels. Hence, we demonstrated that partial voluming with CSF could not explain the FA reduction.

By contrast, the left cortical-spinal tract had substantially increased FA throughout its trajectory compare to the control group. We interpret this increased FA as due to two major factors. The first relates to the tract itself. Ventricular dilatation may lead to stretching, displacement, and resulting increased coherence of the axons in the CST leading to increased FA [32]. The second relates to crossing fibers. A distinctive feature of the left and right CST Tract FA Profiles of this patient is that FA increases near the superior portion where FA decreases for the healthy controls. In the typical subjects, this decrease in FA is the result of crossing fibers from the corpus callosum. In the patient, the amount of crossing fibers is most likely reduced, as indicated by the low callosal FA.

This case demonstrates that AFQ could be applied to a patient with extremely abnormal brain morphology. Tract Profiles provide novel insight into the neurobiology of this patient's white matter injury.

#### Patient 2: Cerebral Palsy

**Figure 6:**
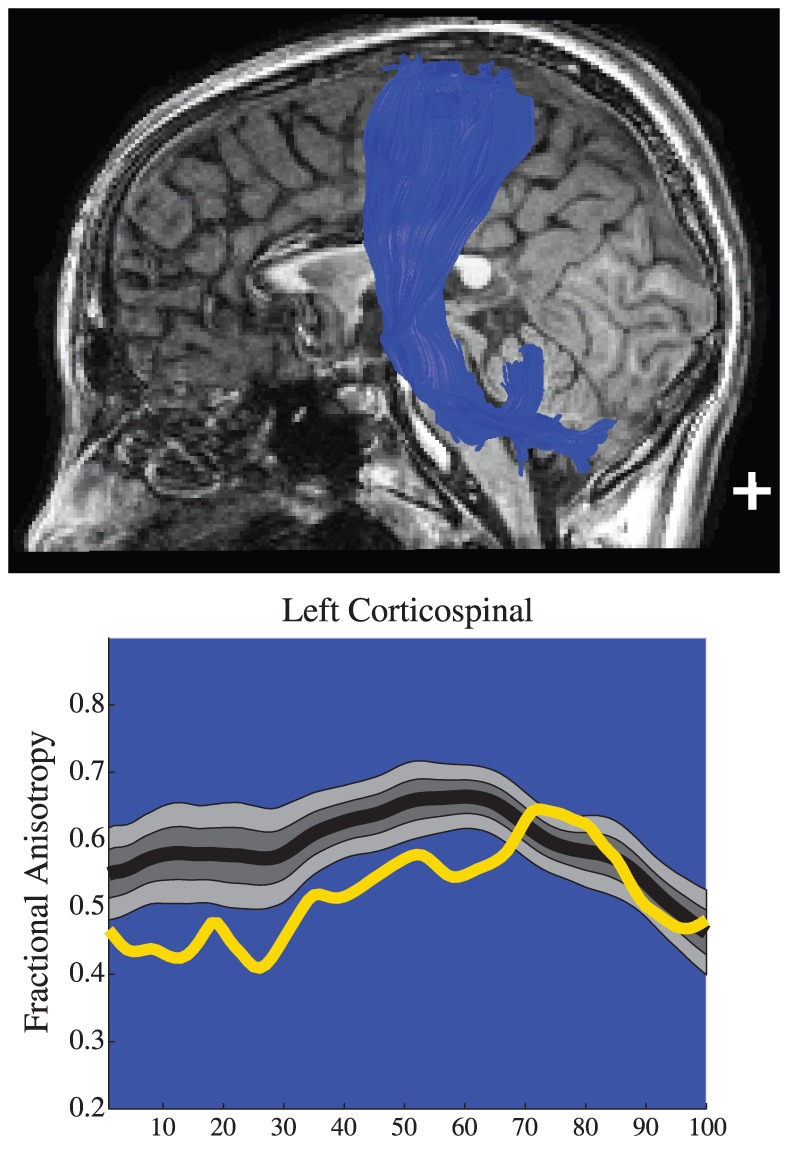
T1 images, tractography results and Tract FA Profiles for Patient 2, a child with cerebral palsy. In the plots, the black line represents the mean FA for typically developing children at 100 points along the tract and light gray region represents the boundaries for the 10^th^ to 90^th^ percentile. The yellow line represents the patient's FA along the tract. The patient has low FA along the corticospinal tract.

Patient 2 was a 12-year-old female born prematurely at 34 weeks gestational age by Cesarean section. At 28 months of age she was diagnosed with mild spastic diplegic cerebral palsy that was more severe on the right side of the body. She was treated with physical and occupational therapy, splinting, and eventually a trial of botulinum toxin injections. T1 weighted anatomical images and the cortico-spinal tract are shown for this child in Figure 6. There was a substantial reduction in FA along the portion of the CST where there are typically no crossing fibers. However in the centrum semioval where crossing fibers reduce FA in healthy control subjects, the patients FA values were within the normal range.

This case demonstrates the sensitivity of AFQ to microstructural differences in a patient without extremely abnormal brain morphology.

### Prediction of reading outcomes in children born preterm

We now demonstrate that AFQ can be used to create behavioral as well as structural Tract Profiles. Most studies that seek to establish the degree of correlation between behavioral function and tissue properties of tracts use the mean value for the entire tract in the calculations. We generate “Behavioral Tract Profiles” by calculating the degree of correlation between behavioral measures and tissue properties at multiple points along the tract. We anticipate that the degree of correlation will vary along the tract.

Reading deficits are common in children born preterm [36]. It is thought that they are related to white matter abnormalities secondary to preterm birth [33]–[35], [45]. We used AFQ to create Behavioral Tract Profiles to evaluate the correlation between localized white matter structure and reading skills in the sample of children born preterm (n = 26) and to compare these correlations to the those of a sample of typically developing children (n = 18).

Single word reading is thought to utilize an interconnected network of brain regions, including the superior temporal gyrus, inferior parietal lobe, and the inferior frontal gyrus. Two main pathways connecting these regions are the arcuate fasciculus and superior longitudinal fasciculus. Previous studies of typically developing children have reported a negative correlation between diffusion properties in the left arcuate fasciculus and phonological processing skills, which are considered essential for reading development [6]. The involvement the arcuate fasciculus in phonological processing is also supported by the analysis of neurological cases [46] and intra-operative micro-stimulation [47]. We contrast the correlations computed for tract average FA versus correlations computed along the Tract FA Profiles for the left arcuate and left SLF in full term and preterm children.

We first used AFQ first to replicate the correlation between reading skills and tract mean diffusion properties of the left arcuate in typically developing full-term children. For the typically developing children we found a significant negative correlation between single word reading skills and tract mean FA of the left arcuate fasciculus (r = −0.40, p = 0.05, one-tailed). The direction and magnitude of this correlation was very similar to the direction and magnitude of the correlation between phonological processing skills and left arcuate fasciculus FA in a previous study of typically developing children (Yeatman et al. 2011). We also found a negative correlation between single word reading skills and tract mean FA of the left SLF (r = −0.30, p = 0.10, one-tailed), a trend that did not reach statistical significance. For the preterm children, we found a significant positive correlation between single word reading skills and tract mean FA of the left arcuate (r = 0.44, p<0.05, 95% CI = 0.22 to 0.64) and the left SLF (r = 0.41, p<0.05, 95% CI = 0.10 to 0.66).

**Figure 7:**
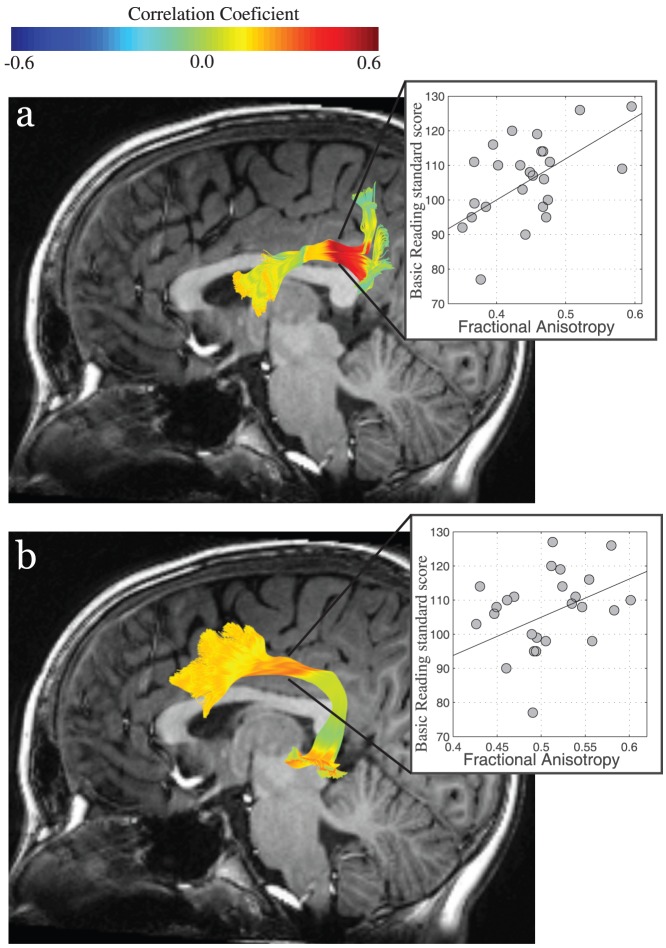
Behavioral Tract Profiles show the correlation between reading skills and FA along the left superior longitudinal fasciculus and left arcuate fasciculus. The correlation between reading skills and FA was computed at each point along the Tract FA Profile for the (a) left superior longitudinal fasciculus and (b) left arcuate fasciculus in the children born preterm. The resulting Behavioral Tract Profile is mapped to the fiber tracts of a single representative subject. Colors correspond to the magnitude of correlation between reading scores and FA at each of 100 equidistant points along the tracts for the children born preterm. The correlations were not uniform along the tracts. Scatter plots show the association between FA (x-axis) and Basic Reading Standard Scores (y-axis) for the point of maximal correlation.

Behavioral Tract Profiles demonstrated that the reading-FA correlations varied significantly along the left arcuate and left SLF. Figure 7 uses a color map to represent the variation in the correlation coefficient at the different locations along the trajectory of the left arcuate and left SLF in children born preterm. The degree of correlation was not uniform. For the left SLF the correlation with basic reading skills was significantly higher for a central portion of the tract approximately 1 cm in length (r = 0.55, p<0.05 corrected, 95% CI = 0.24 to 0.74) than in distal portions of the tract. The correlations ranged from r = 0.1 to r = 0.3 in the regions where fibers branch away from the central section and approached r = 0 near the tract endpoints. The location of highest correlation between arcuate fasciculus FA and single word reading occurred at the same anatomical location as the location of developmental change. This is the location at which the tract is coherently bundled together and does not curve or branch. All the correlations remained significant after controlling for age.

These analyses confirm that the AFQ segmentation can detect previously reported brain-behavior correlations within a sample of typically developing children and detect novel correlations in a sample of patients born preterm. Examining correlations at multiple locations along the trajectory of a fascicle provides superior sensitivity to brain-behavior correlations than does summary measurements. This analysis provides a framework for predicting an individual patient's behavioral outcome based on their deviation from typical diffusion measurements.

## Discussion

We developed and evaluated a novel methodology for automatically identifying fiber tracts and quantifying tissue properties at multiple locations along their trajectories. The resulting Tract Profiles elucidate fundamental properties of white matter tracts in healthy and diseased brains. First, FA values vary substantially within a tract but the shape of the Tract FA Profile is consistent across subjects. Hence the Tract Profile contains information beyond the tract mean. The consistency of Tract Profiles demonstrates the precision of this method for quantifying tissue properties at specific locations on a fiber tract in an individual's brain. Second, Tract Profiles localize developmental changes in FA to specific regions of fiber tracts. FA development is not uniform along the full tract. Third, Tract Profiles can be used to compare individual patients with healthy population norms to elucidate unique features of that patient's clinical condition. Finally, Behavioral Tract Profiles predict variation in behavioral outcomes in children born preterm. FA measurements sampled from specific locations on the left arcuate fasciculus and left superior longitudinal fasciculus correlate with reading proficiency in the preterm children.

Other methods for parameterizing MRI measurements along the trajectory of white matter tracts have been used to study healthy brain anatomy [24], [25], development [26], aging [27], and clinical conditions including epilepsy [28], premature birth [29], [32], neuromyelitis optica [30] and fetal alcohol syndrome [31]. In each of these studies Tract Profiles elucidate white matter characteristics obscured by analysis of tract mean measurements. For example Davis et al. demonstrate that aging does not affect fiber tract diffusion properties uniformly; age related white matter deficits increase gradually, from posterior to anterior, along the length of the uncinate fasciculus and cingulum bundle [27]. Berman et al. demonstrate that the decline in corticospinal tract FA at the level of the centrum semiovale is present in infants at 42 weeks of gestational age but is not present in premature infants at 29 weeks of gestational age [29]. Concha et al. demonstrate that diffusion abnormalities caused by epilepsy are restricted to the temporal lobe portion of fiber tracts though abnormalities in the temporal lobe portion of the arcuate fasciculus are not detected in tract mean measurements [28]. O'Donnell et al. demonstrate that laterality measurements are not uniform along tracts; in this case the arcuate fasciculus is significantly left lateralized at only two specific locations on the tract [25].

Our contribution includes a complete and automated data processing pipeline that runs from raw DTI data to fiber tract identification and Tract Profile quantification for 18 major fiber tracts. In addition we document the white matter features that contribute to the shape of each Tract Profile and propose a framework for applying these methods to the quantification of abnormalities in individual patients. Open-source software for the analysis of Tract Profiles will allow Tract Profiles to be a standard of the field and provide opportunities to systematically compare the advantages of each methodology for computing Tract Profiles. We put the AFQ software in the public domain so that others can use it and/or modify it for their particular purposes.

### Tract Profiles are consistent across subjects

Using AFQ we found that each tract had characteristic peaks and valleys in its Tract FA Profile and these peaks and valleys are at the same locations across healthy and typically developing children (Figure 1 and Figure 2). Many major white matter fascicles can be thought of as highways with distinct entrances and exits where populations of axons join, diverge or cross the main fascicle. Declines in FA indicate locations on the tract with crossing and branching axons, high tract curvature, or intermixing of CSF and gray matter within the same voxels that contain the tract.

Analyzing Tract Profile of diffusion measurements along the trajectory of the tract provides insight into the tissue properties of these localized regions. A tract's profile of FA measurements can be summarized with the population mean and standard deviation at each location of the tract so that an individual can be quantitatively compared to population norms. Changes in FA due to development or disease may reflect different biological processes and have different behavioral implications depending on their location on a tract.

### Developmental changes in FA are localized to specific regions of fiber tracts

AFQ Tract Profiles confirm that FA increases between late childhood and early adolescence for most major white matter tracts [20]. We added new information, that FA changes are localized to specific sub regions of the tract and do not occur along the entire trajectory of a tract. These sub-regions were consistent for each tract in the left and right hemisphere. For example in the frontal lobe portion of the left IFOF, FA was more than 6 standard errors of the mean higher for older children compare to younger children whereas the rest of the tract had nearly equivalent FA for both groups. We think that this large difference reflects developmental changes within distinct populations of axons that comprise the fascicles. This theory is consistent with reports of late maturation of frontal lobe white matter and frontal lobe dependent cognitive skills [41], [42], [48]. Voxel-based analyses have demonstrated a posterior to anterior developmental trajectory where the timing of development depends on the voxels location along the anterior-posterior axis of the brain [48]. We show that this pattern is present at the level of fiber tracts: Not only do frontal lobe tracts develop later, but the anterior portion of large tracts develop later than the posterior portions.

An alternative hypothesis is that the rate of myelination varies along the length of an axon [26], [49]. Averaging FA for the whole tract masks the magnitude and specificity of developmental change.

### Tract Profiles detect neurodevelopmental abnormalities in individual children born preterm

Using AFQ Tract FA Profiles for the analysis of individual clinical cases, we found that Tract FA Profiles are sensitive to white matter abnormalities associated with ventricular dilatation and cerebral palsy. From a clinical perspective, decisions are made at the individual level, taking into account the cognitive, behavioral and neurological characteristics of the patient. It is therefore essential to establish the sensitivity of diffusion imaging to changes within the brains of individual patients before considering applications within the clinic [50]. AFQ Tract Diffusion Profiles are sensitive to white matter abnormalities within an individual's brain and provide quantitative metrics that may aid in clinical decision-making. However establishing the utility of AFQ within the clinic will require rigorous testing of the sensitivity and specificity of these quantitative metrics for specific clinical conditions.

### Localized FA measurements on the arcuate and SLF predict reading skills

We used Behavioral Tract Profiles to investigate the neurobiology of individual differences in reading skills in healthy and injured brains. For typically developing children left arcuate fasciculus FA is negatively correlated with single word reading skills. This finding is in line with previous reports [6]. For children born preterm, left arcuate fasciculus FA and left SLF FA are both positively correlated with single word reading skills. The magnitude of the correlation varies along the trajectory of the tracts, with the largest correlation coefficient occurring along the central portion where fibers are coherently bundled together and oriented anterior-posterior. The location on the tract where the correlation is highest elucidates the potential biological characteristics that underlie the correlation. Within this central portion of the tract there is minimal contamination of FA measurements from crossing and curving fibers and FA values might be more indicative of the organization of axons within the main fascicles than are FA values at other locations.

Diffusion measurements and neurological case studies clearly point to an association between the left arcuate fasciculus and reading related skills [6], [46], [51] for review see [52]. However, children can learn to read without the arcuate fasciculus when damage occurs early in life [53]. Longitudinal and intervention studies are needed to understand how the anatomy of the arcuate fasciculus interacts with reading instruction and reading skills. Future research, with additional quantitative measurements is needed to explain why the FA-reading correlation is negative in typically developing children yet positive in a clinical population of children born preterm.

### Tract Profiles versus voxel based analysis

Automated Fiber Quantification is based on tracking specific fiber groups in individual subjects. We use this approach because the principal alternative, whole-brain voxel-based analyses (VBA), requires co-registering data across subjects and computing statistics at each voxel. Such methods lack the necessary precision, for making inference at the individual level. For example, Hua et al. (2008) identified 20 fiber tracts in 28 individuals based on tractography and co-registered these brains to create a fiber tract probability map. For each tract they quantified the proportion of subjects with fibers in each voxel. There were very few voxels that corresponded to the same tract for more than half the subjects. Voxel-based probability maps can provide a rough guide for where major tracts are likely to be found. However, diffusion differences identified by VBA are likely to include errors from misalignment of structures. Differences between groups may represent analysis of different structures and not necessarily differences localized to a specific white matter tract. The issue of misalignment is particularly problematic for clinical populations where fiber tracts take varying trajectories around injured brain regions. We have demonstrated that in a pediatric, clinical, population with high variability in brain anatomy, AFQ can reliably identify 18 major white matter fascicles and localize abnormalities at specific locations on these fascicles in individual patients.

### Future Directions

The AFQ software is modular and allows users to incorporate new analysis methods and data types. For clinical purposes conventional low b-value DWI data and a tensor model may be optimal because these data are rapidly acquired, have a high signal to noise ratio and are sufficient for the accurate identification of 18 major white matter tracts with AFQ. Newly developed high angular resolution diffusion imaging (HARDI) data acquisition, models and tractography algorithms may provide additional precision particularly for tracts such as the SLF that pass through multiple regions of crossing fibers. However, the benefits of HARDI data for Tract Profiles for will need to be tested in future studies.

AFQ provides a framework for combining quantitative imaging data from multiple modalities. While diffusion imaging is quantitative, diffusion properties are not biologically specific. Future work using quantitative T1 and Proton Density (PD) in combination with DWI-tractography based fiber tract segmentation will elucidate the precise biological underpinnings of neural injuries in clinical conditions including multiple sclerosis.

The AFQ segmentation procedure can be modified to include additional fiber tracts. The vertical occipital fasciculus is a recently characterized fiber bundle that connects to a region in ventral occipital temporal cortex that is essential for skilled reading [54]. Furthermore the SLF has three subcomponents, SLF 1, 2 and 3, that AFQ analyzes as a single fascicle [55]. In our data the distribution of fiber coordinates within the ILF is bimodal suggesting that the typical ILF segmentation convolves two separate fiber bundles that could be separated. These detailed segmentations were beyond the scope of this paper but are targets for future software development within AFQ.

A current limitation of AFQ is that only a central portion of the fiber tract is analyzed. This decision avoids the need for additional coregistration procedures because as we have shown, the central portion is in register across subjects. Some studies suggest that analyzing profiles along the full length of the tract requires manual coregistration of tract landmarks [27], while other studies suggest that re-sampling the tract to an equivalent number of nodes is sufficient [31]. Future releases of AFQ will include an algorithm to automatically identify tract landmarks and align full Tract Profiles across subjects.

### Conclusions

The opportunity to automatically quantify diffusion properties along a tract enriches the understanding of normal and abnormal anatomy. It has increased sensitivity to detection of developmental and clinical changes and increased specificity for the identification of locations of change compare to methods that summarize a whole tract with a single statistic. This methodology appears to offer enormous potential in the clinical setting, particularly in the comparison of individual patients to normative populations by providing quantitative assessments of the individual patients deviation from the norms. We recognize that we are reporting on healthy children and adolescents and only one clinical population. The demonstration that this approach can be applied more generally in clinical research and practice requires investigations of its utility in other clinical populations. To facilitate future studies in different clinical groups we make the software open source and freely available at https://github.com/jyeatman/AFQ with online documentation available at http://white.stanford.edu/newlm/index.php/AFQ.

Further testing of the algorithms is necessary. Ultimately, we hope that rapid accurate methods of white matter characterization can be done at young ages to identify children at risk for neurodevelopmental disorders. The children can then receive appropriate interventions to ameliorate their conditions. These methods may prove sensitive to changes in the diffusion properties as a function of successful intervention, providing evidence of the mechanisms of change with therapy.

## Methods

### Subjects

Participants were 9–16 years old and enrolled in the Palo Alto CA site of a larger multi-site study of prematurity outcomes [36]. This study reports on 48 typically developing control children and 26 age- and gender-matched children born preterm, all of whom underwent MRI scanning at Stanford University. The Stanford University institutional review board approved this study. A parent provided informed consent; children provided assent.

Preterm subjects were born at <36 weeks gestation with birth weight <2500 grams. Controls were born >37 weeks. Exclusion criteria for all participants included seizure disorder; hydrocephalus; receptive vocabulary score <70; sensorineural hearing loss; and non-English speaker. Controls were excluded for identified language, learning, or psychiatric disorders.

Medical complications at birth in the preterm group were: 4 with abnormal findings on head ultrasounds or MRIs (>grade 2 intraventricular hemorrhage, echodensities, or cystic lesions), two with mildly abnormal findings (grade 1 hemorrhage or choroid plexus cyst); 13 had respiratory distress syndrome, five developed bronchopulmonary dysplasia (BPD) or chronic lung disease; none had necrotizing enterocolitis; and two were small for gestational age (<3^rd^ percentile birth weight for gestational age).

### Behavioral Assessment

All 26 of the preterm subjects and 18/48 of the full term control subjects were administered the Woodcock Johnson Basic Reading Skills Composite Index. The Basic Reading Skills Composite Index combines the scores on the Woodcock Johnson Word Identification subtest that assess reading single words and the Word Attack subtest that measures reading of pseudo-words. The mean Basic Reading Skills score was 107 (sd = 9.7) for the full term control subjects and 105 (sd = 12.5) for the preterm subjects.

### Diffusion weighted imaging acquisition and processing

Diffusion weighted imaging (DWI) data were acquired on a 3T Signa Excite (GE Medical Systems, Milwaukee, WI) at Stanford University. A diffusion-weighted, single-shot, spin-echo, echo-planar imaging sequence (TE = 80 ms, TR = 6500 ms, FOV = 240 mm, matrix size = 128×128) was used to acquire 60 2 mm-thick slices in 30 different diffusion directions (b = 900) for a voxel resolution of 2×2×2 mm. The sequence was repeated 4 times and 10 non-diffusion weighted (b = 0) volumes were collected.

Eddy current distortions and subject motion in the diffusion-weighted images were removed by a 14-parameter constrained non-linear co-registration based on the expected pattern of eddy-current distortions given the phase-encode direction of the acquired data [56].

Each diffusion-weighted image was registered to the mean of the (motion-corrected) non-diffusion-weighted (b = 0) images using a two-stage coarse-to-fine approach that maximized the normalized mutual information. The mean of the non-diffusion-weighted images was automatically aligned to the T1 image using a rigid body mutual information algorithm. All raw images from the diffusion sequence were resampled to 2-mm isotropic voxels by combining the motion correction, eddy-current correction, and anatomical alignment transforms into one omnibus transform and resampling the data using a 7th-order b-spline algorithm based on code from SPM5 [57]. An eddy-current intensity correction was applied to the diffusion-weighted images at the resampling stage.

The rotation component of the omnibus coordinate transform was applied to the diffusion-weighting gradient directions to preserve their orientation with respect to the resampled diffusion images. The tensors were then fit using a robust least-squares algorithm designed to remove outliers from the tensor estimation step [58]. We computed the eigenvalue decomposition of the diffusion tensor and the resulting eigenvalues were used to compute the fractional anisotropy (FA), mean diffusivity, (MD), radial diffusivity (RD) and axial diffusivity (AD) [2]. FA is the normalized standard deviation of the three eigenvalues and indicates the degree to which the isodiffusion ellipsoid is anisotropic (i.e., one or two eigenvalues is larger than the mean of all three eigenvalues). MD is the mean of the three eigenvalues, which is equivalent to one-third of the trace of the diffusion tensor. RD is the mean of the second and third eighenvalues. AD is the first eigenvalue.

All the custom image processing software is available as part of our open-source mrDiffusion package available for download from http://white.stanford.edu/software/.

### Automated Fiber Quantification (AFQ)

We developed a software package for the automatic identification and quantification of cerebral white matter pathways that we are making open source and freely available. The methodology and algorithms are described here. The AFQ software can be downloaded from https://github.com/jyeatman/AFQ. Additionally, we are releasing an in-depth users manual that describes the code in more detail and provides a step-by-step guide to data analysis with AFQ. In this manuscript we apply AFQ to quantify diffusion properties of major white matter fascicles. The software was designed with flexibility to allow analysis of other quantitative MRI measurements such quantitative T1, proton density and magnetization transfer.

#### Fiber Tract Identification

**Figure 8:**
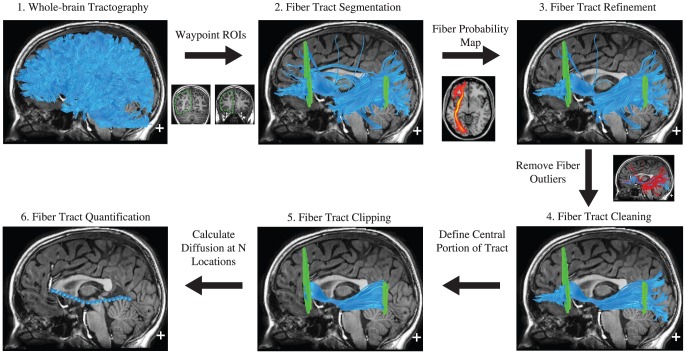
Automated Fiber Quantification (AFQ) procedure for the left hemisphere inferior fronto-occipital fasciculus. (1) Whole brain tractography is initiated from each white matter voxel with fractional anisotropy (FA) >0.3. (2) Fibers that pass through two waypoint regions of interest (ROIs) become candidates for the left IFOF fiber group. (3) Each candidate fiber is then scored based on its similarity to a standard fiber tract probability map. Fibers with high probability scores are retained. (4) Fibers tracts are represented as a 3-dimensional Gaussian distribution and outlier fibers that deviate substantially from the mean position of the tract are removed. (5) The fiber group is clipped to the central portion that spans between the two defining ROIs. (6) The fiber group core is calculated by resampling each fiber into 100 equidistant nodes and calculating the mean location of each node. Diffusion measurements are calculated at each node by taking a weighted average of the FA measurements of each individual fibers diffusion properties at that node. Weights are determined based on the Mahalanobis distance of each fiber node from the fiber core.

AFQ uses a three-step procedure to identify 18 major fiber tracts in an individual's brain. The procedure is based on a combination of the methods described by Hua et al. [59] and Zhang et al. [60]: (1) fiber tractography, (2) waypoint region-of-interest (ROI)-based fiber tract segmentation and (3) fiber tract refinement based on a probabilistic fiber tract atlas. Figure 8 depicts the AFQ analysis pipeline.

Step one, fiber tractography (Figure 8, panel 1): By default this step estimates fiber tracts using a deterministic streamlines tracking algorithm (STT) [8], [9] with a fourth-order Runge–Kutta path integration method and 1-mm fixed-step size. The tracking algorithm is seeded with a white matter mask defined as all the voxels with a fractional anisotropy (FA) value greater than 0.3. A continuous tensor field is estimated with trilinear interpolation of the tensor elements. Starting from initial seed points within the white matter mask, the path integration procedure traces streamlines in both directions along the principal diffusion axes. Individual streamline integration is terminated using two standard criteria: tracking is halted if (1) the FA estimated at the current position is below 0.2 and (2) the minimum angle between the last path segment and next step direction is greater than 30°. This tracking procedure produces a candidate database of fibers for the whole-brain that can then be segmented into anatomically defined fascicles. Note that this step can be done with different fiber orientation estimation methods (tensor, spherical harmonic etc.) and different tractography algorithms.

Step two, fiber tract segmentation (Figure 8, panel 2) is done based on the waypoint ROI procedure described in Wakana et al. [17]. In this procedure fibers are assigned to a particular fiber group if they pass through two waypoint ROIs that define the trajectory of the fascicle. The ROIs are defined in locations that isolate the central portion of the tract where the fibers are coherently bundled together and before they begin diverging towards cortex. Each waypoint ROI was drawn on a group-average DTI data set in MNI space based on the anatomical prescriptions defined in Wakana et al. [17]. The ROIs are transformed into an individual's native space based on an estimated non-linear transformation to the MNI template space [57]. This step is equivalent to the procedure described in Zhang et al. [60], however we use a non-linear transformation instead of a linear transformation. This segmentation procedure defines which fibers are candidates for assignment to a particular fiber group.

Step three, fiber tract refinement (Figure 8, panel 3) is accomplished by comparing each candidate fiber to fiber tract probability maps [59]. Hua et al. [59] created fiber tract probability maps of major fascicles by manually segmenting and coregistering each fiber groups for 28 healthy adult subjects, and calculating for each voxel the proportion of subjects with a given tract in that voxel. We transform the fiber tract probability maps into an individual's native space. Then candidate fibers for a particular fiber group are assigned scores based on the probability values of the voxels they pass through. Candidate fibers that take aberrant trajectories through regions of low probability are discarded. Each fiber in the resulting fiber group passes through the two waypoint ROIs that define the central trajectory of the fascicle and also conform to the shape of the tracts as defined by the fiber tract probability maps.

#### Fiber Tract Cleaning

Tractography may make errors because of noise in the data, regions of complex fiber orientation and ambiguous stopping criteria. The result is that a few fibers may be substantially different from the other fibers in that fiber group. To clean each fiber group into a compact bundle spanning between cortical regions, we implement an iterative procedure that removes fibers that are more than 4 standard deviations above the mean fiber length or that deviate more than 5 standard deviations from the core of the fiber tract (Figure 8, panel 4). To calculate a fiber's distance from the core of the tract we first resample each fiber to 100 equidistant nodes and treat the spread of coordinates at each node as a multivariate Gaussian. The fiber tract core is calculated as the mean of each fibers x, y, z coordinates at each node. The spread of fibers in 3-dimensional space is calculated by computing the covariance between each fiber's x, y, z coordinates at each node. Thus each node on the tract is represented as a mean coordinate, μ, and a 3 by 3 covariance matrix, *S*. For each node on each fiber we then calculate its Mahalanobis distance, D_m_(*x*), from the core of the tract as:
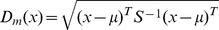
where *x* is a vector containing a fiber node's x, y and z coordinates. The Mahalanobis distance can be interpreted as a z score for a multivariate Gaussian distribution, and corresponds to the probability that a given point belongs to the distribution.

In each iteration, if there are more outliers than would be expected in a Gaussian distribution, those fiber outliers are removed. This process is repeated until there are no more fiber outliers. The resulting fiber groups cohere to the common conception of a fascicle: fibers are coherently bundled together for the central portion of their trajectory before branching toward their cortical destinations.

#### Fiber Tract Quantification

The waypoint ROIs used to identify the fiber groups are defined in planes that are marked by distinct anatomical features and these planes represent equivalent anatomical locations across subjects. The locations of the ROIs isolate the central trajectory of the fascicles. Even though the cortical endpoints of a fascicle typically vary across subjects, the central portion, bounded by the ROIs is generally consistent across individuals. In this report we quantify the diffusion properties of the fiber group along this central portion of the fascicle by clipping each fiber in the fiber group to the portion that spans between the two waypoint ROIs (Figure 8, panel 5) and resampling each fiber to 100 equally spaced nodes. The AFQ software includes options to calculate Tract Profiles for the full tract length or for the region between the defining ROIs. There are benefits to analyzing the full tract length however, it is important to recognize that the distal portions of the tract may not be in register across subjects. Analysis of the full Tract Profile may require additional coregistration procedures.

Diffusion properties are calculated at each node of each fiber using spline interpolation of the diffusion properties: fractional anisotropy FA, mean diffusivity (MD), radial diffusivity (RD) and axial diffusivity (AD). Properties are summarized at each node by taking a weighted average of the diffusion properties at that node on each fiber (Figure 8, panel 6). A fiber's contribution to the average is weighted by the probability that the fiber is a member of the fascicle. This probability is calculated based on the fiber's Mahalanobis distance from the fiber tract core. For example fibers traveling at the core of the fascicle are weighted heavily as these fibers are likely to represent a pure measurement of the tract. Further from the core of the tract diffusion measurements are likely to reflect a mix of white matter and gray matter or white matter and cerebral spinal fluid or white matter from other tracts. The admixing of multiple tissue types within a voxel is known as partial voluming and will bias diffusion measurements. Hence a fiber that diverges from the tract core will not contribute substantially to the tract summary. We summarize each fiber group with a vector of 100 values representing the diffusion properties sampled at equidistant locations along the central portion of the tract. We call this the Tract Profile.

#### Individual and Group Level Inference

Standardized Tract Profiles can be created by calculating the mean and standard deviation of each diffusion property at each node of each tract in a control sample. For our purposes this sample was healthy and typically developing children. We generate confidence intervals for each tract, and can quantify how similar each patient is to the standard Tract Profile.

Univariate statistics such as correlations and T-tests can be calculated point-wise along the Tract Profiles. Given the high degree of correlation between neighboring points on the tract profile each point should not be treated as an independent comparison; hence a Bonferroni correction is overly conservative. We use the permutation based multiple comparison correction described by Nichols and Holmes (2001) to appropriately adjust p-values given the correlation structure of the data [61].

### AFQ produces reliable measures of tract diffusion properties

As a prerequisite for producing and analyzing tract diffusion profiles, we first assessed the reliability of the automated tract segmentation algorithm in identifying the tracts. We reasoned that the algorithm should produce consistent results if multiple scans were obtained for the same individual, akin to test-retest reliability in clinical assessment. Our DWI protocol included four independent repeats of a 30-direction DWI sequence. For each subject, we divided the data into two sets; we averaged scans 1 and 2 as set 1, and scans 3 and 4 as set 2. We then processed each data set with AFQ and extracted the mean FA value for each tract in each individual for the two independent scan sessions. We computed the scan-rescan reliability independently for each tract and found that the median correlation for FA values for each tract from set 1 and set 2 was r = 0.93 with a standard deviation of 0.07. This result demonstrates that the measurements generated by AFQ are highly reliable within an individual across scan sessions. Also note that the correlation reported here represents the reliability of the AFQ analysis for a DWI sequence with 2, 30-direction data sets averaged together rather than the full sequence that we typically use which averages 4, 30-direction data sets. The scan rescan reliability would be even higher if all 4 scans were averaged together.

**Figure 9:**
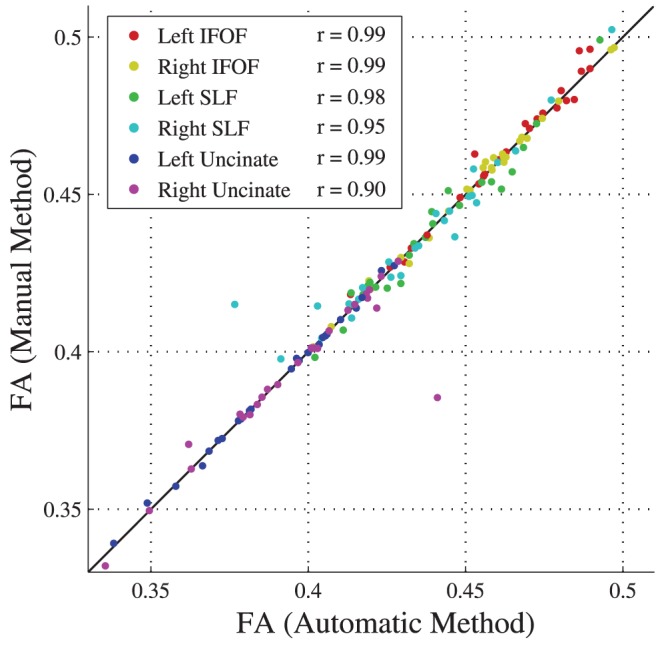
Automated fiber tract segmentation is consistent with manual segmentation. Each point represents the mean FA value for a subject's fiber tract obtained from the automated segmentation (x-axis) and the manual segmentation (y-axis). Mean FA measurements for fiber tracts identified by AFQ are highly correlated with FA measurements from the manual method. The correlation for each tract is in the legend.

As a more demanding measure of reliability, we then compared the mean FA of tracts obtained by two methods–manual segmentation, considered the gold standard for tract identification, (Wakana et al. 2007) and AFQ tract identification. For this analysis we selected six tracts: left and right inferior frontal-occipital fascicle, left and right uncinate fasciculus and left and right superior longitudinal fasciculus. To test the automated method in a clinical sample, we assessed the degree of correlation between tract mean FA measurements from the manual and automated methods in the preterm children. These patients had a range of white matter abnormalities on conventional MRI scans ranging from normal to severe injury, including 3 with severe ventricular dilitation [32]. Correlations between the manual and automated methods were very high for each tract. The median correlation between the FA values obtained from the two methods was r = 0.98 with a standard deviation of 0.04. Figure 9 shows the tract mean FA values obtained from manual segmentation plotted against the values from the AFQ automated segmentation. For nearly every subject the values lie on the identity line demonstrating near perfect correspondence between the methods. Hence The AFQ automated fiber tract segmentation is consistent with the time-consuming manual techniques that have served as the gold standard.

The subject that shows a discrepancy between the manual and automated methods for the right uncinate fasciculus has severe ventricular dilitation. For this subject the automated uncinate ROI placement was imperfect due to extremely abnormal brain shape. Most of the fiber tract segmentations were accurate for these severely abnormal brains, however it is important to manually inspect the ROIs and resulting fiber groups for patients with severe abnormalities because misalignment is possible.
